# Depressive symptoms at age 9–13 and chronic disabling fatigue at age 16: A longitudinal study

**DOI:** 10.1016/j.adolescence.2019.07.014

**Published:** 2019-08

**Authors:** Simon M. Collin, Tom Norris, Carol Joinson, Maria E. Loades, Glyn Lewis, Stephen A. Stansfeld, Esther Crawley

**Affiliations:** a Centre for Academic Child Health, University of Bristol, 1-5 Whiteladies Road, Bristol, BS8 1NU, UK; b School of Sport, Exercise and Health Sciences, Loughborough University, Epinal Way, Loughborough, LE11 3TU, UK; c Department of Psychology, University of Bath, Claverton Down, Bath, BA2 7AY, UK; d Division of Psychiatry, Faculty of Brain Sciences, University College London, London, W1T 7NF, UK; e Wolfson Institute of Preventive Medicine, Barts and The London School of Medicine and Dentistry, Charterhouse Square, London, EC1M 6BQ, UK

**Keywords:** Pediatric, Adolescence, Chronic fatigue, CFS/ME, Mood, ALSPAC

## Abstract

**Introduction:**

We investigated whether depressive symptoms at ages 9–13 years were associated with chronic disabling fatigue (CDF) at age 16 among children in the Avon Longitudinal Study of Parents & Children (ALSPAC) birth cohort.

**Methods:**

Depressive symptoms at ages 9, 10, 11, 12, and 13 years were defined as a child- or parent-completed Short Mood and Feelings Questionnaire (SMFQ) score ≥11 (range 0–26). SMFQ score was also analysed as a continuous exposure. Chronic disabling fatigue at 16 was defined as fatigue of ≥6 months' but <5 years’ duration which prevented school attendance or activities, for which other causes were not identified, and with a Chalder Fatigue Questionnaire score ≥19. Logistic regression was used with multiple imputation to correct for missing data bias. We performed sensitivity analyses in which children who had CDF and depressive symptoms at age 16 were reclassified as not having CDF.

**Results:**

In fully adjusted models using imputed data (N = 13,978), depressive symptoms at ages 9, 11, and 13 years were associated with 2- to 3-fold higher odds of CDF at age 16. Each one-point increase in SMFQ score at ages 9, 10, 11, 12, and 13 years was associated with 6–11% higher odds of CDF at age 16. Depressive symptoms and continuous SMFQ scores at each age were not associated with CDF if the outcome was reclassified to exclude children with comorbid depressive symptoms at age 16.

**Conclusions:**

Depressive symptoms at ages 9–13 were associated with chronic disabling fatigue at age 16, but causality is not certain.

## 1 Introduction

Chronic fatigue syndrome (CFS), also known as ‘ME’ (Myalgic Encephalomyelitis) has two incidence peaks, the first coinciding with adolescence (Bakken et al., 2014). Reported prevalence of clinically-diagnosed CFS/ME (0.1–0.5%) (Chalder, Goodman, Wessely, Hotopf, & Meltzer, 2003; Nijhof et al., 2011; Rimes et al., 2007) is lower than the prevalence (1–3%) of chronic disabling fatigue of at least 6 months' duration estimated from population-based studies (Norris et al., 2017), a discrepancy attributable partly to diagnostic uncertainty among clinicians (Bayliss et al., 2014).

Depressive symptoms occur in up to one third of children with CFS/ME who access specialist services (Bould, Collin, Lewis, Rimes, & Crawley, 2013; Collin, Nuevo, et al., 2015; Loades, Rimes, Ali, Lievesley, & Chalder, 2018), but it is not known to what extent comorbid depression is secondary to CFS/ME (Bould, Lewis, Emond, & Crawley, 2011; Loades, Rimes, Ali, & Chalder, 2019). Whilst low mood is a plausible consequence of the debilitating fatigue that characterizes pediatric CFS/ME (Taylor, Loades, Brigden, Collin, & Crawley, 2017), fatigue is also part of the descriptor of depression.

Longitudinal studies have the potential to elucidate causal relationships, but these have so far provided only slender evidence for depression as a risk factor for later onset of severe fatigue (Bould et al., 2011). We previously used data from the Avon Longitudinal Study of Parents and Children (ALSPAC) birth cohort to show that child psychological factors at age 7–8 years were associated with an increased risk of chronic disabling fatigue (CDF) at age 13, but this association was confounded by maternal mood (Collin, Tilling, et al., 2015). In the present study, we use ALSPAC data to investigate possible associations of depressive symptoms at ages 9, 10, 11, 12, and 13 years with CDF at age 16 years.

## 2 Methods

### 2.1 Participants

ALSPAC is a population-based study, which aims to investigate a wide range of influences on the health and development of children (Boyd et al., 2013). Pregnant women residing in the former Avon Health Authority in south-west England who had an estimated date of delivery between 1 April 1991 and 31 December 1992 were invited to take part, resulting in a cohort of 14,541 pregnancies and 13,978 children alive at 12 months of age (excluding triplets and quads). The ALSPAC study website contains details of all the data that are available through a fully searchable data dictionary (www.bris.ac.uk/alspac/researchers/data-access/data-dictionary/). Ethical approval for the study was obtained from the ALSPAC Ethics and Law Committee (IRB00003312) and the Local Research Ethics Committees.

### 2.2 Measures

#### 2.2.1 Outcome (chronic disabling fatigue at age 16 years)

The method for defining ‘chronic disabling fatigue’ (CDF) at age 16 years (median 16.6, IQR 16.5–16.8 years) has been described previously (Collin et al., 2016). We use the term CDF rather than CFS/ME because children in our study were not examined by a physician. Children were classified as having CDF at age 16 years based on information provided by parents and children. CDF was defined as fatigue (feeling tired or lacking in energy) of >6 months' duration that was associated with absence from full-time school or that had prevented the child from taking part in activities ‘quite a lot’ or ‘a great deal’, excluding fatigue possibly related to sport, snoring and other illnesses. Children could only be classified as having CDF if they scored ≥19 (range 0–33) on the Chalder Fatigue scale. This threshold has 82.4% sensitivity and 86.4% specificity for CFS/ME in adults (Cella & Chalder, 2010; Chalder et al., 1993). Children who met our CDF criteria but who were reported by parents to have had problems with alcohol or drugs (crack, solvents, heroin, or cocaine) or a diagnosis of anorexia nervosa were classified as not having CDF. According to our definitions, the prevalence of CDF of >6 months' duration at age 16 was 1.46% (84/5756). To reduce the likelihood of reverse causality between the primary exposure and the outcome, we excluded children with >5 years' duration of fatigue (11 out of 84 children), hence our analysis was based on n = 73 children with CDF at age 16 years.

#### 2.2.2 Exposures (depressive symptoms at ages 9, 10, 11, 12 and 13 years)

Children completed the Short Mood and Feelings Questionnaire (SMFQ) (Angold et al., 1995) at ages 10 (median 10.6, IQR 10.5–10.8), 12 years (median 12.8, IQR 12.7–12.9 years) and 13 (median 13.8, IQR 13.8–13.9) years, and parents completed the SMFQ when the child was 9 (median 9.6, IQR 9.6–9.7), 11 (median 11.7, IQR 11.7–11.8), and 13 (median 13.1, IQR 13.1–13.2) years old.

The SMFQ is a 13-item scale derived from the 33-items Mood and Feelings Questionnaire (Costello & Angold, 1988). The SMFQ asks about the occurrence of depressive symptoms over the past two weeks. It correlates highly with the Children's Depression Inventory (CDI) (Kovacs, Goldston, & Gatsonis, 1993) and the Diagnostic Interview Schedule for Children (DISC) (Shaffer, Fisher, Lucas, Dulcan, & Schwab-Stone, 2000), and discriminates depressed from non-depressed children in general population samples (Angold, Erkanli, Silberg, Eaves, & Costello, 2002).

We analysed SMFQ as a dichotomized score, defining high levels of depressive symptoms as a score ≥11 and as a continuous score (range 0–26). An SMFQ threshold of 11 had high sensitivity and specificity for an ICD-10 diagnosis of depression at 18 years in the ALSPAC cohort (Turner, Joinson, Peters, Wiles, & Lewis, 2014), and has been used in other population studies (Angold et al., 2002; Thapar & McGuffin, 1998).

### 2.3 Statistical analysis

A set of putative risk factors for pediatric CFS/ME was identified based on our earlier work and a review of the literature (Collin et al., 2016; Collin, Tilling, et al., 2015; Crawley et al., 2012; Norris et al., 2017). To identify associations between our exposures and outcome, we identified potential confounding variables which could potentially bias any observed association between primary exposure and outcome (Mattocks et al., 2008). The first step in this process was to hold consultative meetings with experts in the fields related to the primary (psychological) and other exposures (sleep, physical activity) and specialists in pediatric CFS/ME. Consensus from these meetings was encapsulated in the form of directed acyclic graphs (DAGs). DAGs are causal diagrams which provide a method for formalising and clarifying relationships between variables (Moodie & Stephens, 2010), thereby informing the process of building causal models (Bodnar & Nelson, 2004). DAGs are useful for identifying variables which confound the relationship between two variables, thus providing researchers with a set of variables for which adjustment is necessary (or unnecessary) in order to obtain unbiased estimates of the causal relationship between two variables (Greenland, Pearl, & Robins, 1999). We refer to the final (logistic regression) model comprising the outcome (CDF as a binary variable), the primary exposure (depressive symptoms) and all identified confounders as the ‘analysis model’.

If the analysis model is fitted to a ‘complete case’ dataset, i.e. dropping participants who have missing data for any of the variables in the model, standard errors will be inflated, and bias may arise. If missingness is dependent only on observed data, i.e. date in the analysis model are ‘missing at random’ (MAR), then multiple imputation can be used to correct this bias. Multiple imputation uses a model based on the analysis model plus auxiliary variables which are selected because: a) they are associated with the outcome; b) they are associated with the missingness of the outcome; and c) they do not have too much missing data (to ensure stable imputation models which therefore produce reliable estimates). The number of imputations required to achieve convergence of parameter estimates was determined by checking the estimate of the Monte Carlo error (MCE) in relation to the standard error of the coefficient being estimated. The number of imputations was increased until MCE reached a value which was <10% of the standard error of the estimate (for all models in our study, 50 imputations was sufficient to meet this criteria). The sample size after imputation was N = 13,978, representing children in the ‘core’ ALSPAC sample who were alive at 1 year and who were either a singleton or twin. Multivariable imputation was performed using an imputation sampling method which incorporates all sources of variability and uncertainty in the imputed values (Royston, 2007). The analysis model is then fitted to the imputed datasets, using Rubin's rules to combine the estimates into a single estimate which is unbiased (or less biased) by differential losses to follow up (Rubin, 1987).

Children completed the SMFQ at age 16 (median 16.6, IQR 16.5–16.8) years, and two thirds of ALSPAC children classified with CDF at age 16 had an SMFQ score ≥11 at this age (Collin et al., 2016). On the basis that high levels of fatigue in these children might be secondary to depression, we performed sensitivity analyses in which we reclassified children who had CDF and high levels of depressive symptoms at age 16 as not having CDF.

All analyses were performed using Stata (StataCorp. 2015. Stata Statistical Software: Release 14. College Station, TX: StataCorp LP).

## 3 Results

Data to define CDF at age 16 years were more likely to be missing for children born into lower socioeconomic status households and whose mothers were more likely to experience depression and anxiety (see supplementary file Table S1).

The numbers of participants with outcome and exposure data available for analysis are summarized in Table 1. The proportions of children who had depressive symptoms (SMFQ score ≥11) at each age were higher in those with CDF than without CDF at age 16 (Chi-squared p < 0.001), and median SMFQ scores at each age were higher in children who had CDF at age 16 years compared with children who did not have CDF (Kruskal-Wallis p < 0.001).

**Table 1:** Parent (SMFQ_P_) and child (SMFQ_C_) scores and high levels of depressive symptoms (SMFQ score ≥11) at age 9–16 years in relation to chronic disabling fatigue (CDF) at age 16 years.

		Children without CDF (N = 5683)	Children with CDF (N = 73)
SMFQ_P_ @ 9 yrs (mean 9.7 yrs)	median (IQR)	1 (0–3), n = 4881	4 (2–7), n = 61
	SMFQ≥11	105 (2.15%)	7 (11.5%)
SMFQ_C_ @ 10 yrs (mean 10.6 yrs)	median (IQR)	3 (1–5), n = 4551	5 (3–8), n = 60
	SMFQ≥11	218 (4.79%)	8 (13.3%)
SMFQ_P_ @ 11 yrs (mean 11.7 yrs)	median (IQR)	1 (0–3), n = 4723	3 (1–6), n = 61
	SMFQ≥11	114 (2.41%)	6 (9.84%)
SMFQ_C_ @ 12 yrs (mean 12.8 yrs)	median (IQR)	3 (1–5), n = 4358	4 (2–8), n = 59
	SMFQ≥11	259 (5.94%)	11 (18.6%)
SMFQ_P_ @ 13 yrs (mean 13.2 yrs)	median (IQR)	1 (0–3), n = 4788	3.5 (1–8), n = 60
	SMFQ≥11	137 (2.86%)	10 (16.7%)
SMFQ_C_ @ 13 yrs (mean 13.8 yrs)	median (IQR)	3 (2–7), n = 4114	6 (3–12), n = 54
	SMFQ≥11	410 (9.97%)	19 (35.2%)
SMFQ_P_ @ 16 yrs (mean 16.8 yrs)	median (IQR)	4 (2–7), n = 4370	12 (7–18), n = 35
	SMFQ≥11	610 (14.0%)	21 (60.0%)

In fully adjusted models using imputed data (N = 13,978), the odds of CDF at age 16 in children who had high levels of depressive symptoms at age 9–13 years were 2- to 3-fold higher, compared with children without depressive symptoms at these ages (Table 2). The associations were strongest at ages 9, 11, and 13 years; at ages 10 and 12 years the 95% confidence intervals were consistent with no association. Each one-point increase in SMFQ score at ages 9–13 years was associated with 6–11% higher odds of CDF at age 16 (Table 3).

**Table 2:** Associations (odds ratio (95% CI)) of dichotomous SMFQ scores (score ≥11 indicating high levels of depressive symptoms) at ages 9–13 years with chronic disabling fatigue (CDF) at age 16 years (SMFQ_P_ = completed by parent, SMFQ_C_ = completed by child).

		Raw data	Imputed data^†^	Imputed data^†^ (excluding comorbid depressive symptoms)
Depressive symptoms at age 9 yrs (SMFQ_P_≥11)	Partially adjusted^#^	6.44 (2.74, 15.2)	6.25 (3.10, 12.6)	3.41 (1.10, 10.6)
	Fully adjusted^‡^	–	2.32 (1.02, 5.30)	1.87 (0.56, 6.27)
Depressive symptoms at age 10 yrs (SMFQ_C_≥11)	Partially adjusted^#^	2.67 (1.18, 6.07)	3.14 (1.64, 6.00)	1.58 (0.61, 4.09)
	Fully adjusted^‡^	–	1.70 (0.80, 3.61)	1.11 (0.40, 3.13)
Depressive symptoms at age 11 yrs (SMFQ_P_≥11)	Partially adjusted^#^	4.60 (1.90, 11.2)	6.79 (3.24, 14.2)	3.38 (1.16, 9.85)
	Fully adjusted^‡^	–	2.49 (1.01, 6.10)	1.80 (0.54, 6.01)
Depressive symptoms at age 12 yrs (SMFQ_C_≥11)	Partially adjusted^#^	3.85 (1.93, 7.69)	3.29 (3.14, 3.43)	1.12 (0.39, 3.19)
	Fully adjusted^‡^	–	1.87 (0.93, 3.78)	0.77 (0.26, 2.29)
Depressive symptoms at age 13 yrs (SMFQ_P_≥11)	Partially adjusted^#^	6.48 (3.04, 13.8)	6.86 (3.61, 13.0)	2.71 (1.01, 7.30)
	Fully adjusted^‡^	–	3.03 (1.39, 6.58)	1.51 (0.51, 4.46)
Depressive symptoms at age 13 yrs (SMFQ_C_≥11)	Partially adjusted^#^	5.37 (2.95, 9.79)	3.73 (2.30, 6.05)	1.38 (0.62, 3.05)
	Fully adjusted^‡^	–	2.45 (1.47, 4.07)	1.01 (0.44, 2.30)

† Variables for the multiple imputation were: CDF @ 13y & 16y; sex; night-time sleep duration @ 7y & 9y; BMI @ 7y & 9y; child mood @ 9y, 10y, 11y, 12y, 13y (Short Moods and Feelings Questionnaire score); maternal depression @ 11y (Edinburgh Postnatal Depression Scale); maternal anxiety @ 11y (Crown-Crisp Experiential Index); mean test score for English, Mathematics and Science @ 11y (Key Stage 2 tests); maternal life events score (antenatal); self-esteem @ 8y (Global Self Worth subscale from Harter's Self Perception Profile for Children); ALSPAC family adversity index (antenatal); family adversity index @8-10y; life difficulties, hyperactivity and internalising behaviour @ 11y (Strengths and Difficulties Questionnaire); screen time @ 6y; special school arrangements for emotional/behavioural problems @ 7y; conduct problems @ 8y; duration of breastfeeding; family income @ 4y; maternal childhood socioeconomic status; maternal education; maternal age at birth of child; maternal psychopathology @ 8-10y; experienced bullying @ 8y; days spent outdoors @ 8y; poor concentration at school @ 7y; maternal and paternal BMI @ 8y; internalising behaviour @ 4y (Strengths and Difficulties Questionnaire); early puberty; dietary patterns @ 3y & 10y; physical activity @ 11y & 13y (total accelerometer counts per minute over 7-day period).

# Adjusted for sex and family adversity index (antenatal).

‡ Adjusted for: sex; family adversity index (antenatal); BMI @ 7y & 9y; maternal depression @ 11y; maternal anxiety @ 11y; internalising behaviour @ 4y & 11y; self-esteem @ 8y; child mood @ 9y; early puberty; Key Stage 2 test scores; duration of breastfeeding; family income @ 4y; screen time @ 6y; maternal childhood socioeconomic status; maternal life events score (antenatal); night-time sleep duration @ 8y; conduct problems @ 8y; dietary patterns @ 10y

**Table 3:** Associations (odds ratio (95% CI)) of continuous SMFQ scores at ages 9–13 years with chronic disabling fatigue (CDF) at age 16 years (SMFQ_P_ = completed by parent, SMFQ_C_ = completed by child).

		Raw data	Imputed data^†^	Imputed data^†^ (excluding comorbid depressive symptoms)
SMFQ_P_ at age 9 yrs (mean 9.7 yrs)	Partially adjusted^#^	1.19 (1.13, 1.26)	1.15 (1.10, 1.21)	1.10 (1.02, 1.17)
	Fully adjusted^‡^	–	1.07 (1.01, 1.13)	1.04 (0.97, 1.13)
SMFQ_C_ at age 10 yrs (mean 10.6 yrs)	Partially adjusted^#^	1.13 (1.06, 1.20)	1.12 (1.07, 1.17)	1.06 (1.05, 1.06)
	Fully adjusted^‡^	–	1.06 (1.01, 1.12)	1.02 (0.95, 1.10)
SMFQ_P_ at age 11 yrs (mean 11.7 yrs)	Partially adjusted^#^	1.15 (1.09, 1.22)	1.16 (1.10, 1.21)	1.09 (1.09, 1.10)
	Fully adjusted^‡^	–	1.07 (1.01, 1.14)	1.04 (0.96, 1.12)
SMFQ_C_ at age 12 yrs (mean 12.8 yrs)	Partially adjusted^#^	1.12 (1.05, 1.19)	1.14 (1.09, 1.20)	1.03 (0.96, 1.09)
	Fully adjusted^‡^	–	1.11 (1.07, 1.16)	0.99 (0.92, 1.06)
SMFQ_P_ at age 13 yrs (mean 13.2 yrs)	Partially adjusted^#^	1.15 (1.10, 1.21)	1.15 (1.11, 1.20)	1.09 (1.08, 1.09)
	Fully adjusted^‡^	–	1.09 (1.04, 1.14)	1.04 (0.98, 1.11)
SMFQ_C_ at age 13 yrs (mean 13.8 yrs)	Partially adjusted^#^	1.13 (1.07, 1.20)	1.11 (1.07, 1.16)	1.03 (1.02, 1.03)
	Fully adjusted^‡^	–	1.07 (1.02, 1.12)	0.99 (0.92, 1.07)

† Variables for the multiple imputation were: CDF @ 13y & 16y; sex; nighttime sleep duration @ 7y & 9y; BMI @ 7y & 9y; child mood @ 9y, 10y, 11y, 12y, 13y (Short Moods and Feelings Questionnaire score); maternal depression @ 11y (Edinburgh Postnatal Depression Scale); maternal anxiety @ 11y (Crown-Crisp Experiential Index); mean test score for English, Mathematics and Science @ 11y (Key Stage 2 tests); maternal life events score (antenatal); self-esteem @ 8y (Global Self Worth subscale from Harter's Self Perception Profile for Children); ALSPAC family adversity index (antenatal); family adversity index @8-10y; life difficulties, hyperactivity and internalising behaviour @ 11y (Strengths and Difficulties Questionnaire); screen time @ 6y; special school arrangements for emotional/behavioural problems @ 7y; conduct problems @ 8y; duration of breastfeeding; family income @ 4y; maternal childhood socioeconomic status; maternal education; maternal age at birth of child; maternal psychopathology @ 8-10y; experienced bullying @ 8y; days spent outdoors @ 8y; poor concentration at school @ 7y; maternal and paternal BMI @ 8y; internalising behaviour @ 4y (Strengths and Difficulties Questionnaire); early puberty; dietary patterns @ 3y & 10y; physical activity @ 11y & 13y (total accelerometer counts per minute over 7-day period).

# Adjusted for sex and family adversity index (antenatal).

‡ Adjusted for: sex; family adversity index (antenatal); BMI @ 7y & 9y; maternal depression @ 11y; maternal anxiety @ 11y; internalising behaviour @ 4y & 11y; self-esteem @ 8y; child mood @ 9y; early puberty; Key Stage 2 test scores; duration of breastfeeding; family income @ 4y; screen time @ 6y; maternal childhood socioeconomic status; maternal life events score (antenatal); nighttime sleep duration @ 8y; conduct problems @ 8y; dietary patterns @ 10y

In a sensitivity analysis, SMFQ scores and depressive symptoms at each age were not associated with CDF if the outcome was reclassified to exclude children who had comorbid depressive symptoms (Table 2, Table 3). This was confirmed in a bivariate probit analysis (based on raw data) for depressive symptoms at each age in relation to CDF at age 16 with or without comorbid depressive symptoms (Supplementary File, Table S2).

## 4 Discussion

Depressive symptoms at ages 9–13 years were associated with a 2- to 3-fold increased risk of chronic disabling fatigue at age 16 in this UK birth cohort. Initially large positive associations were attenuated after full adjustment for all potential confounders and were no longer supported by statistical evidence at ages 10 and 12 years. Continuous SMFQ scores at all ages were associated with CDF at age 16, even after adjustment for all potential confounders. However, when we excluded comorbid depressive symptoms from our definition of CDF, all the associations disappeared. Therefore, we cannot assert that our results demonstrate a prospective (causal/developmental) association of depressive symptoms at age 9–13 with an increased risk of onset of CDF in late adolescence. We can only conclude that the observed associations between depressive symptoms and later chronic fatigue reflect (to an unknown extent) ongoing mood disorders, i.e. associations between depressive symptoms at younger and older ages.

### 4.1 Strengths and limitations

The main strength of our study is that it used prospectively collected data from a large population-based birth cohort. Our use of directed acyclic graphs enabled us to examine temporal relationships and to adjust for multiple potential confounders (Moodie & Stephens, 2010). Furthermore, we used multiple imputation to adjust for higher losses to follow up that occur among cohort participants from less affluent social groups (Boyd et al., 2013). The main limitation of our study is that children were not assessed by a physician, which is why we describe our outcome as ‘chronic disabling fatigue’, a proxy for CFS/ME. However, our CDF outcome at age 16 combined parental data with a child-completed Chalder Fatigue Questionnaire (CFQ) score (Collin et al., 2016). We classified children as not having CDF if they had a CFQ score <19, a threshold which has high sensitivity and specificity for CFS/ME in adults (Cella & Chalder, 2010).

Another limitation is that, although we excluded children who had been fatigued for >5 years, this does not eliminate the possibility of overlap between primary exposure and outcome for some of the children. In a UK clinical cohort of pediatric CFS/ME patients, the median (IQR) self-reported duration of illness for patients under 12 years old was 12 (7–23) months and in patients 12–18 years old it was 18 (11–28) months (Collin, Nuevo, et al., 2015). Furthermore, our previous analysis of CDF among children in the ALSPAC cohort at age 13, 16, and 18 years showed that 75% of children recovered after 2–3 years (Norris et al., 2017). This gives us some reassurance that the number of children in the present study whose chronic fatigue was longer than 2 years’ duration is likely to be small, and that the observed associations did not reflect reverse causation, i.e. children with a long history of low mood caused by fatigue. We also note that the observed associations did not become stronger with increasing age at the point when the SMFQ was completed.

Several items on the SMFQ overlap with items characteristic of chronic fatigue, such as restlessness, difficulties with concentration, and tiredness. This could result in inflated depressive symptoms scores in children with fatigue, and it is possible that higher fatigue rather than depressive symptoms is reflected in raised SMFQ scores. Conversely, our sensitivity analysis in which we reclassified all comorbidly fatigued/depressed 16-year-olds will almost certainly underestimate any real association between earlier depression and later fatigue. Our analyses of depressive symptoms and SMFQ scores at ages 9, 10, 11, and 13 are subject to the caveat that the causal diagrams to identify potential confounders were based upon SMFQ recorded at age 12. However, we would expect similar relationships to exist between potential confounders and SMFQ recorded at these other ages.

### 4.2 Our findings on the context of other studies

The main question raised by our finding is “how much (if any) of the observed association between depressive symptoms in early adolescence and increased risk of chronic disabling fatigue later in adolescence can be attributed to a ‘causal’ effect?” or (conversely) “how much of the association simply represents long term low mood with fatigue as a symptom?” Our study is the fourth cohort study that has investigated prospective associations between psychological factors and increased risk of chronic fatigue in children and adolescents (Collin, Tilling, et al., 2015; Rimes et al., 2007; Viner et al., 2008). One of these studies was our previous analysis of ALSPAC data, which showed that child psychological problems (and upsetting life events) at age 7–8 years were associated with an increased risk of CDF at age 13, but evidence for these associations was removed when adjusted for maternal anxiety and depression (Collin, Tilling, et al., 2015). In our analysis of SMFQ at age 12 in relation to CDF at age 16 there was some attenuation when we adjusted for maternal anxiety and depression at age 11 years, and further attenuation (removing completely the effect of SMFQ as a binary exposure) when we further adjusted for a large number (18) of potential confounders that we identified using causal diagrams.

Viner et al. reported 2-fold higher odds (OR = 2.0 (95% CI 1.5, 2.7)) of ‘severe fatigue’ in adolescents age 13–16 years among those who had reported a high level of depressive symptoms (using a lower SMFQ threshold of ≥8) two years previously (Viner et al., 2008). The outcome measure in this study was derived from self-reported (WHO Health Behaviour in School-aged Children questionnaire) data and was defined as extreme tiredness occurring more than once per week (in the last month). Despite the differences in study design, these 2-fold higher odds are consistent with the 2- to 3-fold higher odds that we found for depressive symptoms at age 9–13 years in relation to CDF at age 16. Rimes et al. found that any anxiety or depressive disorder predicted new cases of fatigue among children age 11–15 years, with >4-fold higher odds, albeit over a very short duration of follow-up (4–6 months) and with very small numbers of cases (Rimes et al., 2007).

## 5 Conclusions

We did not find evidence to support a causal association between depressive symptoms at ages 9–13 years and risk of chronic disabling fatigue later in adolescence. The extent to which associations between depressive symptoms and later chronic fatigue reflect ongoing mood disorders, or conversely, the extent to which comorbid depression is secondary to pediatric chronic fatigue requires further study. In the meantime, clinicians working with children with emotional disorders need to be aware of the risk of chronic fatigue during adolescence and should consider liaising with specialist clinical services which have experience of pediatric chronic fatigue.

## Funding

This research was specifically funded by the UK Medical Research Council (grant MR/K020269/1). The UK Medical Research Council and Wellcome (Grant ref: 102215/2/13/2) and the University of Bristol provide core support for ALSPAC. This publication is the work of the authors and Simon Collin and Esther Crawley will serve as guarantors for the contents of this paper. Esther Crawley is funded by an NIHR Senior Research Fellowship (SRF-2013-06-013). Maria Loades is funded by the National Institute for Health Research (Doctoral Research Fellowship, DRF-2016-09-021). Stephen Stansfeld was supported by the National Institute for Health Research (NIHR) Collaboration for Leadership in Applied Health Research and Care (CLAHRC) North Thames at Bart's Health NHS Trust. The views expressed in this publication are those of the authors(s) and not necessarily those of the NHS, the National Institute for Health Research or the Department of Health.

## Authors’ contributions

EC conceived of the study and provided overall supervision; SC and TN performed the analyses; CJ, ML, GL, SS and EC provided subject-matter expertise prior to the analysis and contributed to interpretation of the findings; all authors contributed to drafting and revising the manuscript and approved the final version.
