# Supplementary material for: Depressive symptoms at age 9–13 and chronic disabling fatigue at age 16: A longitudinal study
Source: J Adolesc. 2019 Aug;75:123–9. doi: 10.1016/j.adolescence.2019.07.014 (PMC6706778; doi:10.1016/j.adolescence.2019.07.014)
Supplement: Multimedia component 1 [file mmc1.docx]

**Table S1: Cohort characteristics for children classified with or without chronic disabling fatigue (CDF), or without data for this outcome, at age 16 years**

|  |  | Children without CDF (n=5683) | Children with CDF (n=73) | P-value^*^ | Children missing outcome data (n=8222) | P-value^**^ |
| --- | --- | --- | --- | --- | --- | --- |
| Maternal age (at birth of child) | median (IQR) | 29 (26 - 32) | 31 (28 - 34) | 0.02 | 27 (24 - 30) | <0.001 |
| Maternal education | CSE/Vocational | 1064 (19.3%) | 8 (11.3%) | 0.06 | 2656 (38.9%) | <0.001 |
|  | O-level | 1895 (34.4%) | 23 (32.4%) |  | 2378 (34.8%) |  |
|  | A-level | 1553 (28.2%) | 19 (26.8%) |  | 1222 (17.9%) |  |
|  | Degree | 1005 (18.2%) | 21 (29.6%) |  | 574 (8.4%) |  |
| Maternal EPDS score^***^ | median (IQR) | 4 (1 - 8) | 7 (3 - 12) | 0.004 | 5 (2 - 10) | <0.001 |
| Maternal CCEI score^***^ | median (IQR) | 3 (1 - 6) | 5 (2 - 10) | <0.001 | 3 (1 - 6) | 0.001 |
| Sex of child | Female, n (%) | 2965 (52.2%) | 49 (67.1%) | 0.01 | 3744 (45.5%) | <0.001 |
| SDQ total score (range 0 - 40)^****^ | median (IQR) | 5 (3 - 8), n=4823 | 6 (4 - 11), n=62 | 0.007 | 6 (4 - 10), n=2105 | <0.001 |

* Chi-squared test for proportions, Kruskal-Wallis test for medians, comparing characteristics between children with or without CDF at age 16

** Chi-squared test for proportions, Kruskal-Wallis test for medians, comparing characteristics between children with or without outcome data

*** Edinburgh Postnatal Depression Scale (EPDS) or Crown-Crisp Experiential Index (CCEI) score when child was 11 years old

**** Strengths & Difficulties Questionnaire (SDQ) score when child was 11 years old

**Table S2: Bivariate probit analysis of high levels of depressive symptoms (SMFQ score ≥11) at age 9 to 13 years in relation to chronic disabling fatigue (CDF) with or without comorbid depressive symptoms at age 16 years**^†^

|  | CDF with depressive symptoms at age 16 | CDF only at age 16 (no depressive symptoms) | Depressive symptoms only at age 16 (no CDF) | No CDF and no depressive symptoms at age 16 |
| --- | --- | --- | --- | --- |
|  | Marginal effect (95% CI) ^†^ | Marginal effect (95% CI) ^†^ | Marginal effect (95% CI) ^†^ | Marginal effect (95% CI) ^†^ |
| Depressive symptoms at age 9 yrs (SMFQ_P_≥11) | 0.013 (-0.011, 0.038), p=0.29 | 0.004 (-0.009, 0.016), p=0.58 | 0.136 (0.029, 0.243), p=0.01 | -0.153 (-0.264, -0.043), p=0.007 |
| Depressive symptoms at age 10 yrs (SMFQ_C_≥11) | 0.009 (-0.007, 0.024), p=0.26 | 0.002 (-0.006, 0.009), p=0.66 | 0.110 (0.039, 0.182), p=0.003 | -0.121 (-0.195, -0.047), p=0.001 |
| Depressive symptoms at age 11 yrs (SMFQ_P_≥11) | -0.005 (-0.008, -0.003), p<0.001 | -0.004 (-0.006, -0.001), p=0.001 | 0.132 (0.028, 0.236), p=0.01 | -0.122 (-0.227, -0.019), p=0.02 |
| Depressive symptoms at age 12 yrs (SMFQ_C_≥11) | 0.023 (0.003, 0.069), p=0.02 | 0.004 (-0.004, 0.122), p=0.33 | 0.246 (0.176, 0.317), p<0.001 | -0.274 (-0.346, -0.201), p<0.001 |
| Depressive symptoms at age 13 yrs (SMFQ_P_≥11) | 0.038 (0.005, 0.071), p=0.03 | 0.015 (-0.005, 0.036), p=0.14 | 0.168 (0.074, 0.262), p<0.001 | -0.221 (-0.322, -0.121), p<0.001 |
| Depressive symptoms at age 13 yrs (SMFQ_C_≥11) | 0.026 (0.010, 0.042), p=0.001 | 0.012 (0.001, 0.024), p=0.04 | 0.182 (0.129, 0.235), p<0.001 | -0.221 (-0.277, -0.165), p<0.001 |

† Raw data analysis, adjusted for sex and family adversity index (antenatal). High levels of depressive symptoms at each age except 9 and 10 years were positively associated with ‘CDF with depressive symptoms’ at age 16 but not with ‘CDF without depressive symptoms’ (except at 11 and 13 years). Depressive symptoms at each age were strongly positively associated with ‘depressive symptoms only’ (without CDF) at age 16.
